# Gene Knockout Identification Using an Extension of Bees Hill Flux Balance Analysis

**DOI:** 10.1155/2015/124537

**Published:** 2015-03-22

**Authors:** Yee Wen Choon, Mohd Saberi Mohamad, Safaai Deris, Chuii Khim Chong, Sigeru Omatu, Juan Manuel Corchado

**Affiliations:** ^1^Artificial Intelligence and Bioinformatics Research Group, Faculty of Computing, Universiti Teknologi Malaysia, 81310 Skudai, Johor, Malaysia; ^2^Department of Electronics, Information and Communication Engineering, Osaka Institute of Technology, Osaka 535-8585, Japan; ^3^Biomedical Research Institute of Salamanca/BISITE Research Group, University of Salamanca, 37008 Salamanca, Spain

## Abstract

Microbial strain optimisation for the overproduction of a desired phenotype has been a popular topic in recent years. Gene knockout is a genetic engineering technique that can modify the metabolism of microbial cells to obtain desirable phenotypes. Optimisation algorithms have been developed to identify the effects of gene knockout. However, the complexities of metabolic networks have made the process of identifying the effects of genetic modification on desirable phenotypes challenging. Furthermore, a vast number of reactions in cellular metabolism often lead to a combinatorial problem in obtaining optimal gene knockout. The computational time increases exponentially as the size of the problem increases. This work reports an extension of Bees Hill Flux Balance Analysis (BHFBA) to identify optimal gene knockouts to maximise the production yield of desired phenotypes while sustaining the growth rate. This proposed method functions by integrating OptKnock into BHFBA for validating the results automatically. The results show that the extension of BHFBA is suitable, reliable, and applicable in predicting gene knockout. Through several experiments conducted on *Escherichia coli, Bacillus subtilis*, and *Clostridium thermocellum* as model organisms, extension of BHFBA has shown better performance in terms of computational time, stability, growth rate, and production yield of desired phenotypes.

## 1. Introduction

The rapid development of genetic manipulation techniques has made the alteration of microorganisms for different purposes popular in recent years. Genetic manipulation of microorganisms aims to increase the yields of biocompounds or decrease the production of by-products [1]. The process of developing computational models to simulate the actual processes inside cells is growing rapidly because the models are of central importance to the investigation of general biological functions and applications in the area of biomedicine and biotechnology [2]. In nature, microorganisms evolve by optimising their growth rather than by overproducing specific chemical compounds due to metabolic responses to the history of selective pressures. Hence, retrofitting cellular metabolism is essential to economically developing high-yield cellular production systems. However, data ambiguity due to the complexities of the metabolic networks makes the effects of genetic modification on the desirable phenotypes difficult to predict. Furthermore, the huge number of reactions performed in the course of cellular metabolism often leads to a combinatorial problem in obtaining optimal gene knockout due to the large solution space [3]. The computational time increases exponentially as the size of the problem increases. As mentioned by de Paz et al., the use of computational methods is essential. One of the possible applications is in the use of Artificial Intelligence techniques [4]. In recent years, rational design principles based on genetic engineering have been implemented to retrofit microbial metabolism, a process that is widely known as metabolic engineering. In metabolic engineering, the main objective is to increase target metabolite production through genetic engineering. Gene knockout is one of the most common genetic engineering techniques in which one of an organism's genes is made inoperative. To date, this technology has been successfully applied in many organisms, from unicellular eukaryotes to mammals, including human cells.

Computational algorithms have been developed to identify the gene knockout to obtain improved phenotypes. Burgard et al. developed the first rational modelling framework (known as OptKnock) for introducing a gene knockout, leading to the overproduction of a desired metabolite [5]. OptKnock functions by identifying a set of gene (reaction) deletions to maximise the flux of a desired metabolite without affecting the operation of the internal flux distribution so that growth or another objective function is optimised.

OptKnock uses mixed integer linear programming (MILP) to formulate a bilevel linear optimisation that is a promising method of finding the global optimal solution. OptGene is an extended approach of OptKnock, which formulates the in silico design problem using a Genetic Algorithm (GA) [6]. Metaheuristic methods are capable of producing near-optimal solutions with reasonable computation time. Furthermore, the objective function that can be optimised is flexible. OptGene is developed in two representation schemes: binary or integer. The binary representation is more complex and produces solutions with a larger number of knockouts even though it is closer to the natural evolution of microbial genomes. Although the integer representation results in a more compact genome, it still encounters problems as it needs to define the number of gene knockouts a priori [7]. Hence, Rocha et al. proposed two optimisation algorithms, Simulated Annealing (SA), and Set-based Evolutionary Algorithms (SEAs), to allow the automatic determination of the best number of gene deletions to achieve a given productivity goal. Still, these methods do not guarantee to reach optimal solutions due to their stochastic nature [8]. The computational algorithms discussed in this paper are based on constraint-based models. According to Egen and Lun, to date, more than 50 organism-specific genome-scale models have been developed and used in various applications, and it is believed that constraint-based models can produce more accurate predictions [9].

A hybrid of BA and FBA (BAFBA) was proposed by Choon et al. [10]. BAFBA showed better performance in predicting optimal gene knockout in terms of growth rate and production yield. The concept of BAFBA is based on Bees Algorithm (BA) introduced by Pham et al. [11]. BA is a typical meta-heuristic optimisation approach, which has been applied to various problems, such as controller formation [12], image analysis [13], and job multiobjective optimisation [14]. The concept of BA is based on the intelligent behaviour of honeybees. It locates the most promising solutions and selectively explores their neighbourhoods looking for the global maximum of the objective function. BA is efficient in solving optimisation problems, according to previous studies. Nevertheless, BA is relatively weak in local search activities due to its dependency on random search [15]. BHFBA, a hybrid of Hill climbing and the neighbourhood searching strategy of BAFBA, was proposed to improve the performance of BAFBA by using the Hill climbing algorithm as a promising algorithm in finding the local optimum [16]. In this paper, we propose an extension of BHFBA by integrating OptKnock into BHFBA for validating the results automatically. This paper shows that the extension of BHFBA is not only capable of solving large problems in short computational time but also improves the performance in predicting optimal gene knockout. We also present the results obtained by extension of BHFBA in four case studies, with* E. coli* (*Escherichia coli*)* i*JR904,* B. subtilis* (*Bacillus subtilis*), and* C. thermocellum* (*Clostridium thermocellum*) as the target microorganisms. In addition, we conducted a benchmarking to test the performance of the hybrid Bee algorithm and Hill Climbing algorithm.

This paper is organised as follows. First, the materials and experimental setup are described. Then, the problem formulation is introduced, and the details of the BAFBA and the extension of BHFBA are described. Next, experimental results are presented. Then, the obtained results are discussed, reviewing the contributions of this work. Finally, this paper is summarised by providing the main conclusion and addresses future developments.

## 2. Materials and Methods

### 2.1. Materials

In this study, we used* E. coli*,* B. subtilis*, and* C. thermocellum* models to test the operation of the extension of BHFBA.* E. coli i*JR904 (http://bigg.ucsd.edu/) was used to test the operation of BAFBA [17]. The* E. coli* model contains 904 genes, 931 unique biochemical reactions, and 761 metabolites. We used* E. coli i*JR904 in this work to test the reliability of BHFBA because this model was used in previous studies [5, 6, 10]. This model is preprocessed through several steps based on biological assumptions and computational approaches before it was applied. This results in the reduction of the size of the model to 667 reactions. The second model is* B. subtilis i*Bsu1103 [18] (http://genomebiology.com/content/supplementary/gb-2009-10-6-r69-s4.xml), which includes 1437 reactions associated with 1103 genes. We preprocessed this model to reduce the size to 763 reactions. The last model is* C. thermocellum* (ATCC 27405) iSR432 model [19] (http://www.biomedcentral.com/content/supplementary/1752-0509-4-31-s3.xml), which contains 577 reactions, representing the function of 432 genes. The preprocessing of this model reduced the size to 351 reactions. The growth rate and BPCY were used in this work. The unit for growth rate is hour^−1^, while the unit for BPCY is milligram (gram-glucose.hour)^−1^.

We compared the results with those of previous reports in the literature [5, 6, 10]. The experiments were conducted on a 2.3 GHz Intel Core i7 processor and 8 GB RAM workstation. We carry out 100 individual runs in the experiment to test the efficiency of BHFBA, and the result shown is the best result among the runs.

### 2.2. Method

#### 2.2.1. Problem Formulation

The problem of identifying optimal gene knockout from biological models can be formulated as follows. Suppose that a model that contains the stoichiometric matrix **S** provides the linear relationship of the model between the flux rates of the reactions (**v**) and the derivatives of the reactant concentrations (**x**). The matrix is a constant, while the flux vector is a variable. Assume that there are *m* reactants and *n* reactions between them.

Flux vector:

(1) $$\begin{array}{c} v={\left(v_{1},v_{2},\ldots ,v_{n}\right)}^{T}. \end{array}$$

Concentration vector:

(2) $$\begin{array}{c} x={\left(x_{1},x_{2},\ldots ,x_{m}\right)}^{T}. \end{array}$$

Dynamic mass balance equation:

(3) $$\begin{array}{c} \frac{dX}{dt}=Sv, \end{array}$$

where **T** represents the time.

The chemical elements, ionic charge, and biochemical moieties must be balanced in the stoichiometric matrix. The objective is to find the optimal gene knockout to improve the product yields of industrially important chemicals while sustaining the growth rate of the microorganism. This is commonly performed using linear programming, defined as follows:

(4) maximiseMcTxsubject  toMSv=0,maximiseMlowerbound≤x≤upperbound,

where **v** represents the vector of fluxes and **S** is the stoichiometric matrix. The expression (**c** ^**T**^ **x**) to be maximised or minimised is known as the objective function, where **c** is a vector of weights, indicating how much each reaction contributes to the objective function. The inequalities of the lower bound and upper bound define the maximal rates of flux for every reaction corresponding to the columns of the stoichiometric matrix.

#### 2.2.2. A Hybrid of BA and FBA (BAFBA)

**Figure 1:**
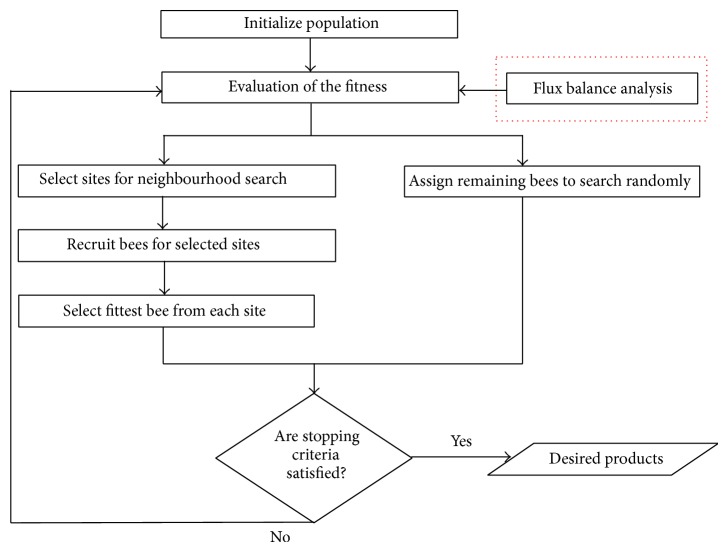
BAFBA flowchart.* Note*. Red-dotted box is Flux Balance Analysis which is hybridized into standard BA as an objective function in order to predict the effect of gene knockout.

Figure 1 shows the flow of the BAFBA. The BAFBA is initialised by mimicking a population of bees. In identifying gene knockout, a bee is represented by a binary variable to indicate the absence or the presence of genes in the reaction. In this study, the BAFBA is started with the bees being placed randomly in the search space. The fitness of the sites visited by the bees is evaluated using the FBA. Bees with the highest fitness would be denoted as “selected bees” and the sites they visited would be chosen for a neighbourhood search. A small amount of “selected bees” was expected to encourage local exploitation. After many tests, we found that an appropriate maximum “selected bees” was (1/4) ×  *n*. We chose and limited the amount of selected bees within the range [1, (1/4) ×  *n*] to prevent the selection of too many sites for a neighbourhood search. Each bee was required to go through this repetitive local search neighbourhood procedure until the best possible answer was obtained. Meanwhile, the remaining bees were assigned randomly to search for new potential solutions.

Before attempting to propose the extension of BHFBA, it is crucial to find the limitations of the BAFBA [10] and BHFBA [16]. The dependence of BA on random search makes it relatively weak in local search activities, and it suffers from slow convergence due to the repetitive iteration of the algorithm. The repetition of unnecessary similar process in the neighbourhood search causes additional computational time in generating the solution. In addition, the results need to be validated manually.

#### 2.2.3. An Extension of Bees Hill Flux Balance Analysis (BHFBA)

**Figure 2:**
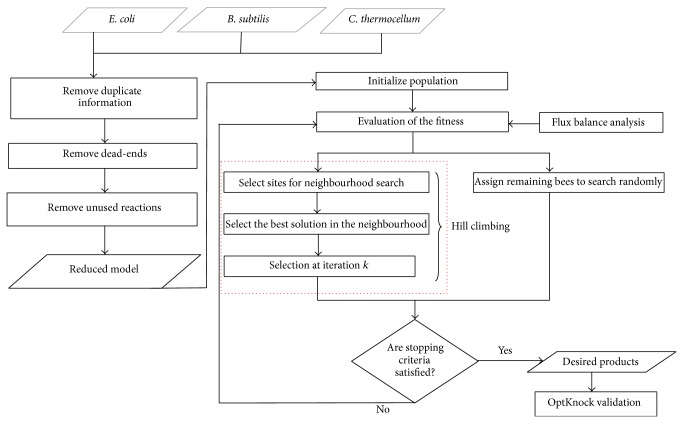
BHFBA flowchart.* Note*. Red-dotted box is Hill Climbing algorithm which is hybridized into BAFBA in order to improve the local search performance of BAFBA.

In this paper, we propose the extension of BHFBA to identify optimal gene knockout. It is proposed to overcome the limitations of BAFBA and previous reports. The extension of BHFBA in our work differs from the BAFBA in local search activities and in validating the results. The extension of BHFBA improves the algorithm by hybridising Hill Climbing algorithm with BAFBA and by integrating OptKnock into BHFBA.  Figure 2 shows the overall framework of BHFBA. Important steps are explained in the following subsections.

**Figure 3:**
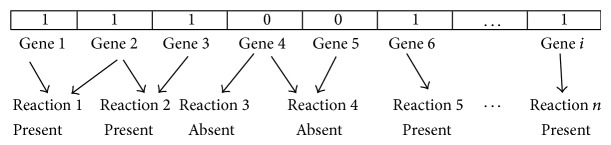
Bee representation of metabolic genotype.

*Bee Representation of Metabolic Genotype*. One or more genes can be involved in each reaction in a metabolic model. In this paper, each of those genes is represented by a binary variable, where 0 represents the absence of the gene and 1 represents the presence of the gene in the reaction. These variables form a “bee” representing a specific mutant that lacks some metabolic reactions when compared with the wild type (Figure 3).

*Initialisation of the Population*. The algorithm starts with an initial population of *n* scout bees. Each bee is initialised as follows. Assume a reaction with *n* genes. Bees in the population are initialised by randomly setting the present or absent status of each gene. Initialisation of the population is performed randomly so that all bees in the population have an equal chance of being selected. The result might not truly reflect the population if it is performed with a bias setting.

*Evaluation of the Fitness (Flux Balance Analysis)*. Each site is given a fitness score that determines whether more bees should be recruited or whether the site should be abandoned. Here, we use the FBA to calculate the fitness score for each site (see (4)). In this paper, maximisation of growth is applied. After maximising cellular growth, mutants with growth rate higher than 0.1 continue the process by maximising the desired product flux at fixed optimal cellular growth value. Hence, we enhance the yield of our desired products at a fixed optimal cellular growth. The production yield is the maximum amount of product that can be generated per unit of substrate. The following shows the calculation for production yield:

(5) Production  yield=production  rate  production  mmol/gmconsumption  rate  substrate  mmol/gm,

where mmol is millimole and gm is gram.

We used biomass-product coupled yield (BPCY) as the fitness score in this work. According to Soons et al., metabolic networks can function in living cells under various biological objectives depending on the relevant organism and its genetic and environmental context. However, biological objectives are only applicable for analysing a number of organisms in terms of microbial metabolic engineering. It is desirable to couple the formation of the desired product to growth [20]. The calculation for BPCY is as follows:

(6) BPCY=production  yield  mmol/gm ×growth  rate  mmol·hr/gm·hr,

where mmol is millimole, hr is hour, and gm is gram.

**Figure 4:**
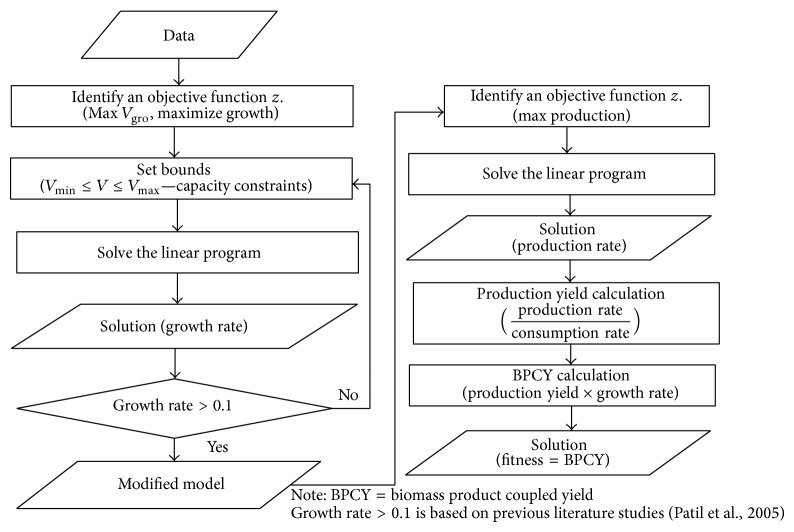
The flow for calculating fitness function.

The flow of calculating the fitness function is shown in Figure 4.

*Neighbourhood Search (Hill Climbing Algorithm)*. This algorithm carries out neighbourhood searches in favoured sites (*m*) using the Hill Climbing algorithm. Hill climbing is an iterative algorithm that starts with an arbitrary solution to a problem and then attempts to find a better solution by incrementally changing a single element of the solution. In this paper, the initial solution is the *m* favoured sites from the population initialised with the BA. The algorithm starts with the solution and makes small improvements to it by adding or reducing a bee to the sites. We define the value of initial size of patches (ngh) and use the value to update the site (*m*) identified in the previous step to search the neighbourhood area. In this paper, *m* is equal to 15 and ngh is equal to 30. The values are obtained by conducting a small number of trials with the range of 10 to 25 and 20 to 35, respectively. This step is important because there might be better solutions in the neighbourhood than the original solution.

*Random Assignment and Termination*. The remaining bees in the population are sent randomly around the search space to scout for new feasible solutions. This step is performed randomly to avoid overlooking potential results that are not in the initial range. These steps are repeated until either the maximum loop value is met or the fitness function has converged. In the end, the colony has two components in its new population—representatives from each selected patch and other scout bees assigned to perform random searches.

**Figure 5:**
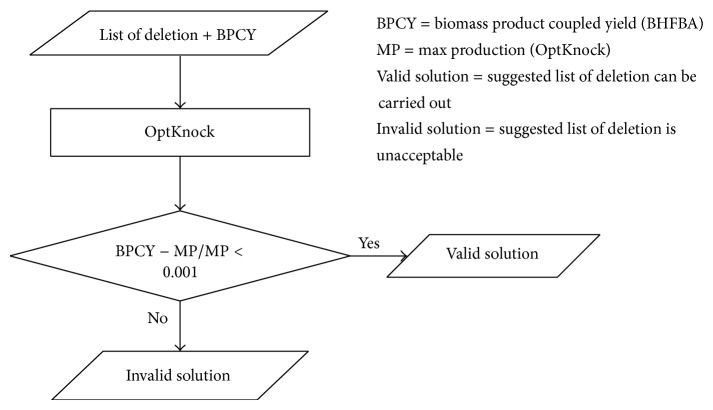
The flow of OptKnock validation.

*OptKnock Validation*. Originally, the result from BHFBA is solely validated through literature. In this paper, we use OptKnock to evaluate the result obtained from the BHFBA. OptKnock is used to evaluate the results by using the list of gene deletions from the BHFBA. If the difference between the BPCY obtained from the BHFBA and the maximum production rate obtained by OptKnock is less than 0.001, the list is considered a valid solution. This saves the biologists' time as they can consider only the valid solution to carry out their laboratory experiments. The list of genes in this paper is the best valid solution among 100 individual runs. After the validation, most of the knockout genes are proven to be related in improving the desired products through literature.  Figure 5 shows the flow of the validation.

## 3. Results and Discussion

### 3.1. Benchmark Functions

In this paper, we propose an improved method, extension of BHFBA, to test the performance of the BHFBA. For evaluation, we conduct a benchmarking analysis. However, benchmark functions can only be tested on BH and BA because FBA is an objective function. Hence, we test the benchmark functions on BH and BA in this study. Because BA is used to look for a maximum, the functions are inverted before the algorithm is applied. The De Jong, Martin and Gaddy, Schwefel, and Griewangk functions are used in this study. These functions are a set of common parametric test problems. The simplest test function is De Jong. It is continuous, convex, and unimodal. Martin and Gaddy function is a unimodal function. The Schwefel function is complex, with many local minima. Lastly, Griewangk function has many widespread local minima. However, the location of the minima is regularly distributed. We carry out 100 individual runs to test BH and BA.

Table 1 shows the mathematical representation of the functions.  Table 2 shows the mean and standard deviation (STD) of the De Jong, Martin and Gaddy, Schwefel, and Griewangk functions tested on both the original BA and BH. The results show that BH performs better than the BA. All functions had a low STD, indicating that the result from each run is very close to the mean. In conclusion, the stability of the proposed method is high given that the difference in the result of each individual run is small. In addition, the means for both algorithms are similar, indicating that BH is indeed reliable because the results obtained from BH are consistent with the results from previous reports.

### 3.2. Production of Succinic Acid and Lactic Acid in* E. coli*

In this paper, the extension of BHFBA is compared with the previous works: BAFBA, SA + FBA, and the conventional OptKnock. Tables 3 and 4 summarise the results obtained from the BHFBA for succinic acid and lactic acid production in* E. coli.* As shown in the results, this method produces better results than the previous studies in terms of growth rate and BPCY and is able to identify potential genes that can be removed.

**Figure 6:**
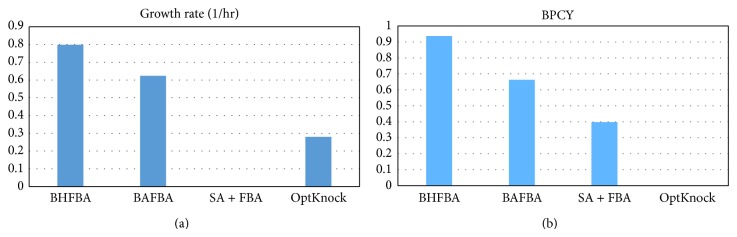
Comparison between different methods for growth rate and BPCY of succinic acid by* E. coli*.* Note*. BPCY is in gram (gram-glucose·hour)^−1^.

Table 3 shows that the extension of BHFBA performs better than those proposed in previous studies with a growth rate of 0.7988 and BPCY of 0.93656. In addition, Figure 6 shows that the extension of BHFBA obtained the highest value for both growth rate and BPCY among the other methods tested. Knocking out succinate dehydrogenase (SUCD1i) interrupted the conversion of succinic acid to fumarate. By eliminating the conversion of succinic acid to fumarate, the production yield of succinic acid is improved. Next, phosphotransacetylase (PTAr) is removed. According to Burgard et al. [5], these mutants can grow anaerobically on glucose by producing lactate. In the next step, ribulose-5-phosphate-3-epimerase (RPE) is suggested to be knocked out. This knockout involves the inflow reaction of ammonium. As stated in Bohl et al., the utilisation of nitrate as the electron acceptor and ammonium source under anaerobic conditions can improve succinate production [21].

**Figure 7:**
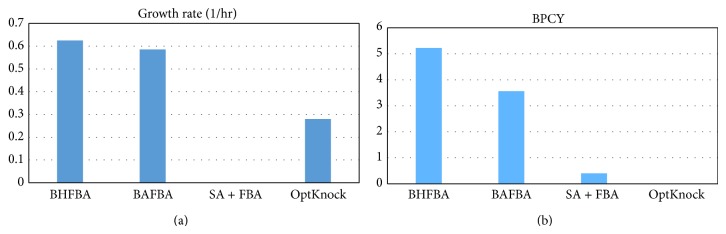
Comparison between different methods for production of lactic acid by* E. coli. Note*. BPCY is in gram (gram-glucose·hour)^−1^.

Table 4 shows the results of the extension of BHFBA and previous works. The extension of BHFBA resulted in a better growth rate and BPCY than the previous works, which are 0.62501 and 5.2241, respectively.  Figure 7 shows the comparison among the methods of producing lactic acid in* E. coli*. The extension of BHFBA shows a drastic difference in the value of BPCY and a small improvement in the growth rate. The deletion of fructose bisphosphatase and phosphoglycerate kinase decreased the efficiency of gluconeogenesis, which resulted in an increased concentration of phosphoenolpyruvate. Phosphoenolpyruvate was then converted into pyruvate and then lactic acid. Knocking out acetaldehyde dehydrogenase, which catalyses the conversion of acetaldehyde into acetic acid, eliminated the competing product, acetic acid. In consequence, the yield of lactic acid is improved.

### 3.3. Production of Ethanol by* B. subtilis*

We applied the BAFBA to* B. subtilis* and* C. thermocellum* to identify the optimal gene knockouts to improve the production of ethanol. Ethanol is a volatile, flammable, and colourless liquid, and it is a promising biofuel. Ethanol is currently used as an alternative fuel for gasoline worldwide. Hence, ethanol is a good case study here.

**Figure 8:**
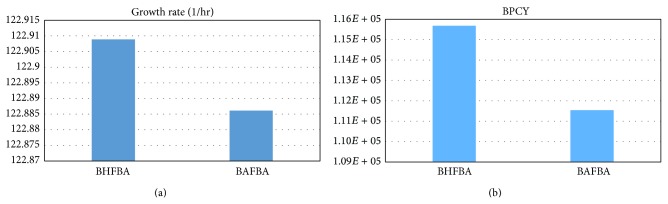
Comparison between different methods for growth rate and BPCY of ethanol by* B. subtilis*.* Note*. BPCY is in gram (gram-glucose·hour)^−1^.

Table 5 shows the results of the extension of BHFBA and previous works. The extension of BHFBA obtained a growth rate and BPCY of 122.9089 and 1.15680*e* + 05, respectively. In the experiment by Kim et al., deletion of NADH-dependent glycerol-3-phosphate dehydrogenase 1 (GPDH) resulted in a slight improvement in ethanol yield. As stated in Kim et al., lactate dehydrogenase (LDH_L) plays a key role in the fermentative metabolism in the metabolic engineering of* B. subtilis* for ethanol production. The deletion of LDH_L inhibited the conversion from pyruvate to lactate, so more pyruvate was decarboxylated to acetaldehyde and further converted to ethanol [22].  Figure 8 shows the comparison between different methods in terms of the growth rate and BPCY of ethanol; the extension of BHFBA generates better results in terms of both growth rate and BPCY compared to BAFBA.

### 3.4. Production of Ethanol by* C. thermocellum*

**Figure 9:**
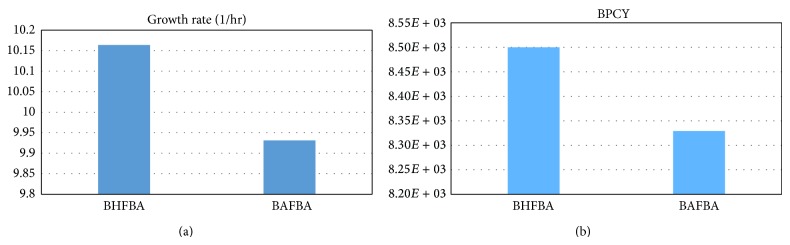
Comparison between different methods for production of ethanol by* C. thermocellum. Note*. BPCY is in gram (gram-glucose·hour)^−1^.

Table 6 shows the results of the extension of BHFBA and previous methods to enhance the production of ethanol in* C. thermocellum*. The extension of BHFBA provides a better result for the* C. thermocellum* model with a growth rate of 10.1637 and a BPCY of 8.5*e* + 003.  Figure 9 shows the comparison between different methods in terms of the growth rate and BPCY of ethanol. The extension of BHFBA results in a higher value of both the growth rate and BPCY than the BAFBA. The list of knockout genes includes nucleoside-diphosphate kinase (NDPK5), glycerol-3-phosphatedehydrogenase (G3PD1) and phosphate acetyltransferase (PTAr). According Roberts et al., the deletion of PTA2, PTAr, PPAKr, and ACKr is expected to increase the lower and upper bounds of ethanol secretion relative to wild-type ethanol secretion [19]. The result indicated that deletion of one of these reactions should force an increase in ethanol production. As mentioned in Kim et al., deletion of NADH-dependent glycerol-3-phosphate dehydrogenase (G3PD1) can slightly improve ethanol production [22]. However, there is still no direct evidence for a unique effect of NDPK5 on ethanol levels in* C. thermocellum*. However, NDPK5 catalyses the reaction in which the terminal phosphate of a nucleoside-triphosphate is transferred to a nucleoside-diphosphate. According to Lu et al., NDPK5 is not essential for growth, but mutants display a mutator phenotype [23].

### 3.5. Computational Time

Table 7 shows a comparison of the computational time required for the extension of BHFBA and BAFBA with 1000 iterations. The average computational time for the extension of BHFBA improved by 69%, 69%, and 72% compared to the BAFBA result for 1000 iterations, respectively.

### 3.6. Discussion

As seen in the results, both the extension of BHFBA and BH performed better than other algorithms. It can be concluded that the ability of the Hill Climbing algorithm to find local optimum improved the performance of the original BA. The original BA has a problem with repetitive iterations of the algorithm in local search, where each bee continues to search until the best possible answer is reached. Our proposed extension of BHFBA solves the problem by implementing Hill Climbing algorithm in the local search and improved the algorithm by integrating OptKnock. The Hill Climbing algorithm is a powerful local search algorithm that attempts to find the best solution by incrementally changing a single element of the solution until no further improvements can be found. The search process is recorded so that the process is not repeated. Furthermore, one of the advantages of the Hill Climbing algorithm is that it can return a valid solution even if it is interrupted at any time before it ends. OptKnock is widely used for* in silico *metabolic engineering. It has been proven that it can produce promising simulated results and help in the experiments.

## 4. Conclusions and Future Works

It is crucial to develop more accurate and efficient modelling and optimisation methods in metabolic engineering because they will have a significant impact on commercialised biotechnology engineering, which will lead to substantial economic gains in the production of pharmaceuticals, fuels, and food ingredients. In this paper, the extension of BHFBA is proposed for use in predicting optimal sets of gene deletions to maximise the production of the desired metabolite. The extension of BHFBA improves the performance of the BAFBA by implementing the Hill Climbing algorithm, which is a promising algorithm for finding local optimum. It is extended by integrating OptKnock into BHFBA. Experimental results with* E. coli i*JR904,* B. subtilis,* and* C. thermocellum* showed that extension of BHFBA is effective in generating optimal solutions for gene knockout prediction; therefore, it is a useful tool in metabolic engineering. In the future because biological models incorporate a set of parameters that represent the physical properties of real biological systems, it is advisable to extend the capability of the parameter estimation method in dealing with the structural nonidentifiability problem. This is because the problem often involves prior knowledge of the structure of the model, which can lead to more discoveries while selecting possible routes of the pathways that are particularly important in the field of bioengineering [24].

## Figures and Tables

**Table 1:** Mathematical representation of De Jong, Martin and Gaddy, Schwefel, and Griewangk functions.

Name	Mathematical representation
De Jong	max⁡F = (3905.93) − 100(*x*_1_^2^−*x*_2_)^2^ − (1−*x*_1_)^2^

Martin and Gaddy	$\min F\;={\left(x_{1}-x_{2}\right)}^{2}+\;{\left(\frac{x_{1}\;+\;x_{2}\;-\;10}{\;3}\right)}^{2}$

Schwefel	$\min F\;=\;418.9829*n\;+\;\overset{d}{\underset{i=1}{\sum }}{-x}_{i}*\sin\left(\sqrt{\left|x_{i}\right|}\right)$

Griewangk	$\min F=\frac{1}{\left(0.1+\sum_{i=1}^{n}\left({x_{i}}^{2}/4000\right)-\prod_{i=1}^{n}\cos\left(\left(x_{i}/\sqrt{i}\right)+1\right)\right)}$

**Table 2:** Obtained fitness value of all benchmark functions.

Function	Mean		STD	
	BA	BH	BA	BH
De Jong	3.91*e* + 03	3.90*e* + 03	0.000504	4.79**e** − 13
Martin and Gaddy	11.1083	11.1111	0.002797	0
Schwefel	8.38*e* + 02	8.38*e* + 2	2.205*e* − 05	0
Griewangk	−0.5263	−0.5263	5.76765*e* − 09	0

*Note*. The bold numbers represent the best result.

**Table 3:** Comparison between different methods for production of Succinic acid by *E. coli*.

Method	Growth rate (1/hr)	BPCY	List of knockout genes
BHFBA	**0.7988**	**0.93656**	PTAr^**^, RPE, SUCD1i
BAFBA [9]	0.62404	0.66306	FUM, PTAr^**^, TPI^**^
SA + FBA [5]	N/A	0.39850	ACLD19^*^, DRPA, GLYCDx, F6PA, TPI^**^, LDH_D2, EDA, TKT2, LDH_D-
OptKnock [3]	0.28	N/A	ACKr, PTAr^**^, ACALD^*^

*Note*. The bold numbers represent the best result. N/A: not applicable. ^**^Common genes in either 2 methods. BPCY is in gram (gram-glucose·hour)^−1^.

**Table 4:** Comparison between different methods for production of Lactic acid by *E. coli*.

Method	Growth rate (1/hr)	BPCY	List of knockout genes
BHFBA	**0.62501**	**5.2241**	FBP, PGK, ACALD^**^
BAFBA [9]	0.58586	3.5656	GAPD, L_LACD2, PTAr^**^
SA + FBA [5]	N/A	0.39850	ACLD19^**^, DRPA, GLYCDx, F6PA, TPI, LDH_D2, EDA, TKT2, LDH_D-
OptKnock [3]	0.28	N/A	ACKr, PTAr^**^, ACALD^**^

*Note*. The bold numbers represent the best result. N/A: not applicable. ^**^Common genes in either 2 methods. BPCY is in gram (gram-glucose·hour)^−1^.

**Table 5:** Comparison between different methods for growth rate and BPCY of ethanol by *B. subtilis*.

Method	Growth rate (1/hr)	BPCY	List of knockout genes
BHFBA	**122.9089**	1.15680**e** + 05	ALAD_L^*^, GPDH, LDH_L^*^
BAFBA [9]	122.8861	1.1154*e* + 05	ALAD_L^*^, LDH_L^*^, XYLI1, inosose 2,3-dehydratase

*Note*. The bold numbers represent the best result. N/A: not applicable. ^*^Common genes for all methods. BPCY is in gram (gram-glucose·hour)^−1^.

**Table 6:** Result of implementation of different knockout for production of Ethanol in *C. thermocellum*.

Method	Growth rate (1/hr)	BPCY	List of knockout genes
BHFBA	**10.1637**	8.5**e** + 003	G3PD1^*^, NDPK5, PTAr^*^
BAFBA [9]	9.9313	8.329*e* + 003	MDH, G3PD1^*^, PTAr^*^

*Note*. The bold numbers represent the best result. N/A: not applicable. ^*^Common genes for all methods. BPCY is in gram (gram-glucose·hour)^−1^.

**Table 7:** Comparison between average computational time of BHFBA and BAFBA for 1000 iterations.

Model	Method	Computation time (seconds)
*E. coli *	BHFBA	**3223**
	BAFBA [9]	10253
	OptKnock [3]	N/A
	SA + FBA [5]	N/A

*B. subtilis *	BHFBA	**7028**
	BAFBA [9]	22515
	OptKnock [3]	N/A
	SA + FBA [5]	N/A

*C. thermocellum *	BHFBA	**2880**
	BAFBA [9]	10282
	OptKnock [3]	N/A
	SA + FBA [5]	N/A

*Note*. The bold numbers represent the best result. N/A represents that the results are not reported in literature.
